# Antihypertensive Effect of Perla and Esmeralda Barley (*Hordeum vulgare* L.) Sprouts in an Induction Model with L-NAME In Vivo

**DOI:** 10.3390/metabo14120678

**Published:** 2024-12-03

**Authors:** Abigail García-Castro, Alma D. Román-Gutiérrez, Fabiola A. Guzmán-Ortiz, Raquel Cariño-Cortés

**Affiliations:** 1 Academic Area of Chemistry, Autonomous University of the State of Hidalgo, Pachuca-Tulancingo Highway Km 4.5, City of Knowledge, Col. Carboneras, Mineral de la Reforma 42184, Hidalgo, Mexico; ga185930@uaeh.edu.mx; 2 CONAHCYT-Universidad Autónoma del Estado de Hidalgo, Carretera Pachuca-Tulancingo Km 4.5, Ciudad del Conocimiento, Col. Carboneras, Mineral de la Reforma 42184, Hidalgo, Mexico; fabiola_guzman@uaeh.edu.mx; 3 Academic Area of Medicine, Institute of Health Sciences, Autonomous University of the State of Hidalgo, Eliseo Ramírez Ulloa 400, Doctores Pachuca, Pachuca 42090, Hidalgo, Mexico

**Keywords:** antihypertensive action, Perla and Esmeralda barley sprouts, *Hordeum vulgare* L. sprouts, L-NAME, in vivo

## Abstract

**Background:** Hypertension is one of the leading causes of premature death worldwide. Despite advances in conventional treatments, there remains a significant need for more effective and natural alternatives to control hypertension. In this context, sprouted barley extracts have emerged as a potential therapeutic option. This study presents the evaluation of the bioactive properties of extracts from two varieties of barley germinated for different periods (3, 5, and 7 days), focusing on their potential to regulate blood pressure mechanisms. **Objectives/Methods:** The main objective was to assess the effects of these extracts on blood pressure regulation in N(ω)-Nitro-L-Arginine Methyl Ester (L-NAME)-induced hypertensive rats. Renal (creatinine, urea, uric acid, and total protein) and endothelial (NOx levels) function, angiotensin-converting enzyme (ACE) I and II activity, and histopathological effects on heart and kidney tissues were evaluated. **Results:** In particular, Esmeralda barley extract demonstrated 83% inhibition of ACE activity in vitro. Furthermore, the combined administration of sprouted barley extract (SBE) and captopril significantly reduced blood pressure and ACE I and II activity by 22%, 81%, and 76%, respectively, after 3, 5, and 7 days of germination. The treatment also led to reductions in protein, creatinine, uric acid, and urea levels by 3%, 38%, 42%, and 48%, respectively, along with a 66% increase in plasma NO concentrations. **Conclusions:** This study highlights the bioactive properties of barley extracts with different germination times, emphasizing their potential health benefits as a more effective alternative to conventional antihypertensive therapies.

## 1. Introduction

Hypertension remains a critical global health concern, affecting over 1.28 billion individuals worldwide and showing a marked increase in prevalence. This rise presents a challenge, especially in low- and middle-income countries with limited access to healthcare [1]. The condition is closely linked to cardiovascular diseases (CVD), such as strokes, renal dysfunction, cardiac hypertrophy, myocardial infarction, and cardiac failure, hence significantly contributing to premature mortality. Patients with hypertension have a high risk of mortality (>7.5 million deaths per year), hospitalization, and reduced quality of life [1,2,3].

Persistent hypertension accelerates endothelial dysfunction and organ damage, further highlighting the need for prompt and sustained control of blood pressure. Early-onset hypertension has been associated with a higher lifetime risk of cardiovascular events and mortality [4]. The need for timely intervention in younger populations is vital to reduce long-term health risks. While antihypertensive therapies are known to lower blood pressure effectively, less than 20% of hypertensive patients achieve proper control, with significant regional disparities. Non-adherence to treatment due to complex regimens and side effects remains a key barrier to effective management [5].

Blood pressure regulation is influenced by the Renin–Angiotensin–Aldosterone System (RAAS), defined as a hormonal system critical for maintaining electrolyte and fluid balance [6]. Moreover, a deficiency in endothelial nitric oxide (NO) is linked to the overproduction of reactive oxygen species (ROS), known as a key factor in the pathogenesis of hypertension [7]. The increase in ROS triggers vasoconstriction, elevating blood pressure [8]. Among the most common treatments are drug therapies for hypertension, such as angiotensin-converting enzyme (ACE) inhibitors, angiotensin II receptor blockers, calcium channel blockers, and diuretics [9]. However, drug therapies are associated with adverse side effects, such as dizziness, dry cough, and kidney dysfunction, leading to an increased interest in lifestyle modifications and natural alternatives for managing hypertension [10,11]. Barley (*Hordeum vulgare* L.), belonging to the family Poaceae (Gramineae), is one of the Neolithic founder crops of Old World agriculture. It is a flowering plant belonging to the family Poaceae or Gramineae (herbs) that is cultivated in temperate climates across the world at 350–4050 m above sea level [12]. Barley constitutes the fourth most important grain crop in the world after wheat, rice, and maize. Barley grain is used as livestock feed and forage, malt beverages, human food, and soil improvement and has medicinal value, but is barely considered as a highly needed crop of the present era [13,14].

Most research has focused on grains and barley leaves has investigated various polyphenol compounds with antioxidants, antiadipogenic and antiobesity, hypolipidemic, antidepressant, immunomodulatory, and antiproliferative activities [12,14,15]. Barley also plays a significant role in the food and brewing industries, particularly in malt production, where germination is essential [16]. Furthermore, the polyphenols and flavonoids contained in germinated cereals boost protection and resist oxidative stress, providing antioxidant activity [17,18]. Germination enhances the digestibility and bioavailability of nutrients. Therefore, germination is an environmentally friendly, convenient, and economical biochemical process [19]. As of now, germinated barley extracts have not yet been widely exploited on an industrial scale specifically for nutraceutical applications. The seed germination process contributes to significant changes in bioactive compounds and their antioxidant activity, resulting in a possible interaction with some biological processes such as ACE inhibition and free radical scavenging [20,21]. Additionally, gamma-aminobutyric acid (GABA), a neurotransmitter, is present in germinated barley and exhibits antihypertensive effects by relaxing blood vessels [22]. Despite existing studies on the benefits of whole grains and sprouted seeds, limited research addresses germinated barley.

Some data on specific varieties such as Esmeralda and Perla and their effects on metabolic alterations are still in the early stages. Furthermore, previous studies have indicated differences in secondary metabolite proportions between these two varieties, highlighting the importance of evaluating the impact of germination on hypertension [17]. Therefore, this study aimed to assess the impact of sprouted barley extracts from two barley varieties, germinated for 3, 5, and 7 days, on biomarkers of blood pressure regulation in vitro and in vivo.

## 2. Materials and Methods

### 2.1. Reagents

N(ω)-Nitro-L-Arginine Methyl Ester (L-NAME), angiotensin-converting enzyme (ACE) from rabbit lung, and Hippuryl-Histidyl-Leucine (HHL) were obtained from Sigma-Aldrich, Saint-Louis, MO, USA. Captopril was purified to 95–98% purity. All other reagents were of analytical grade, sourced from Merck, Darmstadt, Germany and SPINREACT^®^, Girona, Spain.

### 2.2. Preparation of Barley Sprouts Extracts

Barley grains (*Hordeum vulgare* L.) were collected in Apan, Hidalgo, in October 2021. The seeds were washed, disinfected with a 0.05% sodium hypochlorite solution, and rinsed. Extracts were obtained according to a previously described procedure with some modifications [16,17]. Briefly, barley grains were soaked in water for 24 h and placed onto trays in a humidity chamber at 24 °C and 60–70% relative humidity for germination. Non-sprouted and sprouted grains were used at 3, 5, and 7 days. The sprouted barley was oven-dried (Barnstead Lab-Line, ThermoFisher Sci., Waltham, MA, USA) at 50–55 °C until less than 5% moisture was achieved, then ground and sifted to a particle size of 0.2 mm. Fifty grams of flour were mixed with 200 mL of water at 45 °C, maintaining the temperature in an oven until 70 °C. Afterward, 100 mL of water at 70 °C was added, and this temperature was maintained for 1 h, followed by a 10 min cooling period. The mixture was adjusted to 450 g, centrifuged (Termo IEC Centra GP8R San Diego, CA, USA), and the supernatants were lyophilized (816-333-8811 Labconco, Kansas City, MO, USA) at −62 °C and 0.040 mBar. An average of 20 g of lyophilized (Labconco, Kansas City, MO, USA) extract was obtained for every 400 mL of aqueous extract, which was then stored in a vacuum-sealed container in the freezer.

### 2.3. Evaluation of ACE Activity In Vitro

The antihypertensive activity of barley extracts was determined using the modified method of Cushman and Cheung (1971) [23]. The release of hippuric acid (HA) from the Hippuryl-Histidyl-Leucine (HHL) substrate (H1635 Sigma-Aldrich, USA) was measured. First, 20 μL of ACE solution (2.5 mU/mL) (A6778 Sigma-Aldrich, USA) was mixed with 40 μL of barley extracts (10 mg/g) or 0.01 mg/mL of captopril and incubated at 37 °C for 5 min. Then, 100 μL of HHL (5 mM) was added and incubated at 37 °C for 30 min. The reaction was stopped with 150 μL of HCl (1 M) (7728C J.T.Baker, Phillipsburg, NJ, USA), and HA was extracted by adding 1000 μL of ethyl acetate (319902, Merck), vortexing for 20 s (M37615 Thermo, IA, USA), and centrifuging at 4000 rpm for 12 min. Then, 750 μL of the organic phase was boiled (92 °C) to remove ethyl acetate and concentrate HA, followed by the addition of 800 μL of double-distilled water and vortexing for 20 s.

The absorbance was measured at 228 nm. The percentage of ACE inhibition was calculated using the following equation:

ACE inhibition (%) = ((A sample-Ablank)/(Acontrol-Ablank sample)) × 100(1)

### 2.4. Experimental Animals

Male Wistar rats (weighing 200–220 g, aged 8–10 weeks) were used, supplied by the Autonomous University of the State of Hidalgo. All procedures complied with ethical guidelines approved by the Institutional Ethics Committee (Approval No. CICUAL/004/2022). The rats were housed under controlled conditions (22 ± 2 °C, 12:12 h light–dark cycle), with free access to food and water. Handling was performed following NOM-062-ZOO-1999 guidelines for the care and use of experimental animals.

#### 2.4.1. Experimental Design

After one week of acclimatization, 56 rats were randomly divided into 7 groups (*n* = 8). Group 1 (G1) received a standard diet, while Group 2 (G2) was administered with L-NAME (40 mg/kg/day) in drinking water to induce hypertension. Group 3 (G3) was treated with both L-NAME (40 mg/kg/day) and captopril (5 mg/kg/day).

Four additional groups were treated with barley sprout extracts (SBE): Group 4 (G4) received L-NAME plus 500 mg/kg/day of Esmeralda SBE; Group 5 (G5) was treated with L-NAME plus 500 mg/kg/day of Perla SBE; Group 6 (G6) received L-NAME plus a combination of 250 mg/kg/day of each SBE; Group 7 (G7) was treated with L-NAME and Esmeralda SBE plus a low dose of captopril (250 mg/kg/day and 2.5 mg/kg/day, respectively). Treatments were administered via intragastric cannula over 35 days.

#### 2.4.2. Blood Pressure Measurement

Systolic and diastolic blood pressure were measured weekly using a tail-cuff method with the CODA™ blood pressure monitor (Kent Scientific Corp., Torrington, CT, USA). Rats were pre-trained (5 acclimatization cycles/session) to reduce stress and measurements (10 cycles) were taken in a temperature-controlled room (30–32 °C) following acclimatization cycles. Results were expressed in mm/Hg.

### 2.5. Biochemical Tests

#### 2.5.1. Sample Collection and Tissue Preparation

After 35 days of treatment, rats were euthanized by cervical dislocation. Blood was drawn through a cardiac puncture and centrifuged at 3500 rpm for 10 min to obtain plasma for biochemical analyses. Kidneys (500 mg) were homogenized in 0.1 M TRIS-HCl (pH 7.4), centrifuged at 10,000× *g* for 10 min, and the supernatants were used to evaluate angiotensin-converting enzyme (ACE) activity. Portions of the kidney, heart, and liver were washed with a 0.9% saline solution and preserved in 10% formalin for histopathological analysis.

#### 2.5.2. NOx Quantification

Nitrite/nitrate levels in plasma were quantified using the Griess reaction [24]. Nitrate was reduced to nitrite through a 30 min incubation with nitrate reductase and NADPH. For the colorimetric reaction, 100 μL of Griess reagent was added to 50 μL of the diluted sample (1:10) and deionized water, making a final volume of 200 μL. Nitrite was detected as an azole dye visible at 540 nm. Nitrite/nitrate levels were estimated from a standard curve (3–200 μMol/L) and expressed as μMol/L.

#### 2.5.3. Renal Function Markers

Plasma urea, uric acid, and creatinine were estimated according to the manual provided by commercial diagnostic kits (SPINREACT^®^, Spain) based on the Fawcett and J. E. Scott (1960), Caraway (1955), and Jaffe M (1886), respectively [25,26]. The plasma glucose level was determined from an enzymatic reaction using the method described by Trinder (1969) [27] in which glucose oxidase catalyzes the oxidation of glucose to gluconic acid and was measured at 492 nm. The total protein concentration in plasma was determined from the Biuret colorimetric method at an absorbance of 540 nm. Commercial diagnostic kits (SPINREACT^®^, Spain) were used. All results were expressed as mg/dL.

#### 2.5.4. ACE I and ACE II Quantification

Plasma ACE I concentration was measured using a fluorometric assay kit (Sigma-Aldrich, St. Louis, MO, USA) based on the cleavage of a synthetic fluorogenic peptide, with fluorescence proportional to ACE activity (Ex/Em = 320/405 nm). Results were expressed as mU/mL. Kidney ACE II concentration was determined using a similar kit, where 500 mg of kidney tissue was homogenized to assess ACE II’s ability to cleave a synthetic peptide 7-metaxicomarin-4-acetic acid (MCA), releasing a free fluorophore (Ex/Em = 320/420 nm). The results were expressed as mU/mg tissue.

### 2.6. Histopathological Analysis

Samples were fixed at 10% formalin (2106-03 J.TBaker, Phillipsburg, NJ, USA), then dehydrated with ethanol, clarified with xylene, and enclosed with paraffin. Then, 5 μm slices were made, stained with hematoxylin-eosin, and examined with a high-resolution (40×) light microscope (DM500 Leica Microsystems, Wetzlar, Germany).

### 2.7. Statistical Analysis

Data were expressed as the mean ± SD for six determinations and analyzed using one-way ANOVA. Tukey’s test was applied to compare means at a 95% confidence level. To evaluate the relationships between bioactive compounds and ACE inhibitory activity, a Spearman’s test was performed. All statistical analyses were performed using Minitab 19.2.

## 3. Results

### 3.1. Effect of Barley Extracts on ACE Inhibition In Vitro

**Figure 1:**
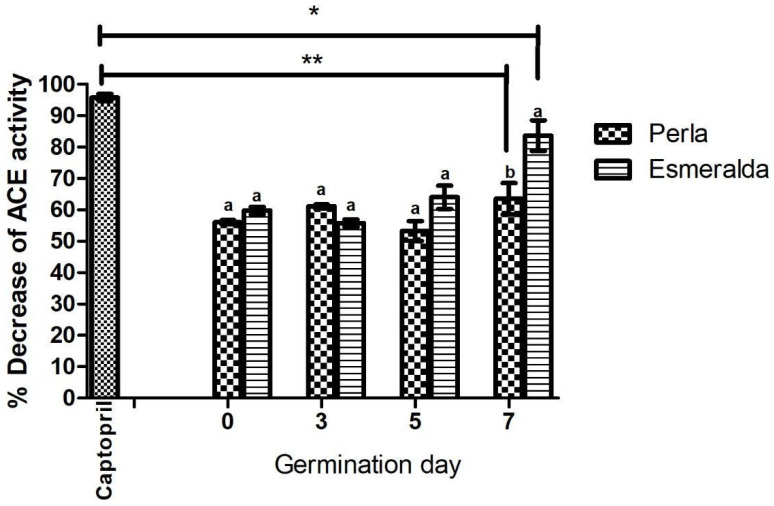
Decrease in a ngiotensin-converting e nzyme activity (percent). Bars represent the mean ± SD. The significance levels are represented by the value of *p* < 0.05 (* *p* < 0.05 and ** *p* < 0.0001) compared to the control group (c aptopril). Different letters indicate significant differences between varieties and the same day of germination. ANOVA followed by a Tukey test was performed.

Figure 1 shows the ACE inhibition activity of captopril and barley sprout extracts (SBE) from Perla and Esmeralda varieties. Captopril at 0.01 mg/mL inhibited 95% of ACE activity, whereas Perla and Esmeralda SBEs showed inhibition between 56 and 63% and 59 and 83%, respectively. Statistically significant differences were observed between all SBE treatments and captopril (*p* < 0.05). The seven-day germinated SBEs of both varieties exhibited increased ACE inhibition, leading to their selection for in vivo administration.

### 3.2. Renal Function and Nitric Oxide Concentration

Table 1 details the effects of SBE on glucose, renal markers, and plasma NOx. No significant differences in glucose were observed between the control and L-NAME groups, though glucose levels rose significantly in G3 (21%) and G6 (29%) (*p* < 0.05). Barley extract germination, associated with higher oligosaccharides, likely contributed to these increases [17].

Regarding renal function, creatinine levels increased by 21% with L-NAME but were significantly improved by 55% with Esmeralda SBE (G4) (*p* < 0.05). Urea and uric acid levels also were observed to decrease by 42% and 48% in G6 and G7, respectively. These results are consistent with other studies that have demonstrated the effects of plant extracts on kidney function. It has been reported that peptides derived from plant proteins can reduce creatinine and urea levels in chronic kidney disease [28]. Furthermore, studies regarding the administration of quercetin and other flavonoids present in barley extracts have shown antioxidant and anti-inflammatory effects that contribute to kidney protection and improvement of glomerular filtration [29]. The study found that endothelial function (NOx) decreased by 37% (*p* < 0.05) in G2 when compared to G1 (Table 1). However, the administration of barley extracts in G7 for 5 weeks demonstrated a significant protection of renal function and increased plasma NOx by 67% (*p* < 0.05), reversing L-NAME-induced declines in NOx concentration. Kumar et al. (2011) [30] reported that induction with L-NAME in rats reduced nitric oxide concentration by up to 60%.

### 3.3. Effect of Extracts of Barley Sprouts on Blood Pressure in Rats

SBEs reduced systolic and diastolic blood pressure (BP) in hypertensive rats induced by L-NAME, as shown in Table 2. Captopril treatment decreased BP by 18%, while SBE administration (G4 and G5) led to BP reductions of 9% and 12%, respectively, compared to G2. The SBE–captopril combination in G7 achieved the most significant BP reduction (22%) by week 3 (*p* < 0.05).

### 3.4. Effects of SBE on ACE I and ACE II Activity In Vivo

**Figure 2:**
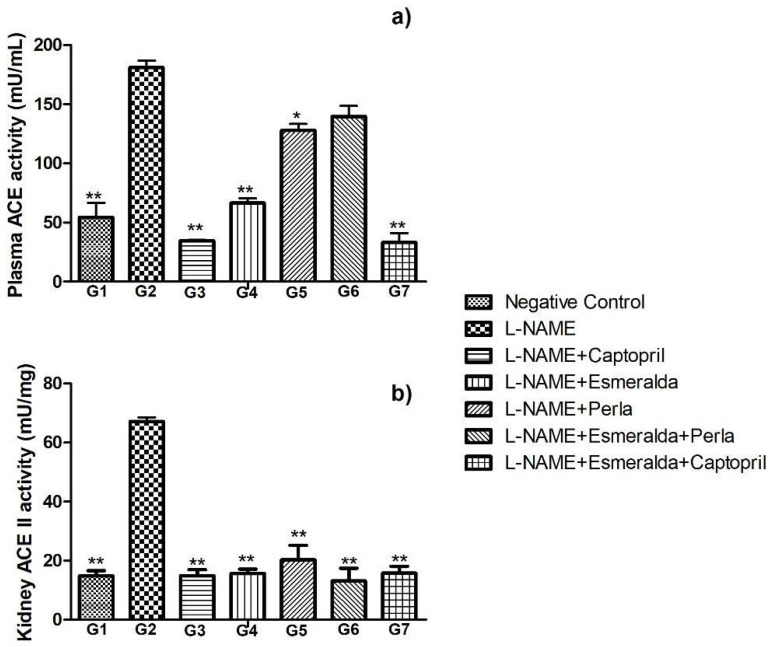
(**a**) Effects of barley extracts and captopril supplementation on serum ACE I activity. (**b**) Effects of barley extracts and captopril supplementation on kidney ACE II activity. Each bar represents mean ± SD. Significance levels indicated by *p* < 0.05 (* *p* < 0.05 and ** *p* < 0.0001) when compared with L-NAME group (ANOVA followed by Tukey test).

Figure 2a,b shows changes in ACE I activity in plasma and ACE II in kidney homogenate. Basal ACE I activity in plasma was 54.4 mU/mL (G1), which increased by 233% after L-NAME administration (G2). Captopril treatment (G3) significantly reduced ACE I activity by 80.8% (*p* < 0.0005). Groups G4 and G5 also showed reductions of 63.3% and 29.3%, respectively. Esmeralda (G4) was selected for combination with captopril (G7) due to its significant reduction in ACE I activity. All other treatments significantly decreased ACE I activity (*p* < 0.05), except for G6, where no significant difference was observed (*p* > 0.05). G7 reduced ACE I activity by 81.6%, similar to G3 (*p* > 0.05).

Figure 2b illustrates changes in ACE II activity in the kidney. Compared to G2, all treatment groups significantly decreased ACE II activity (*p* < 0.0005). Baseline ACE II activity in G1 was 14.8 mU/mg, while G2 experienced a 353% increase. Groups treated with SBE (G4–G6) and the SBE–captopril combination (G7) demonstrated a 76.5% to 80% reduction in ACE II activity compared to G2. This reduction brought ACE II activity levels close to those of the standard diet group (G1) (*p* < 0.05). The BSEs, particularly the Esmeralda variety, significantly reduced the activity of ACE I and II in the plasma and kidney of rats hypertensive with L-NAME, as shown in Figure 2a,b.

### 3.5. Target Organs Histopathology

**Figure 3:**
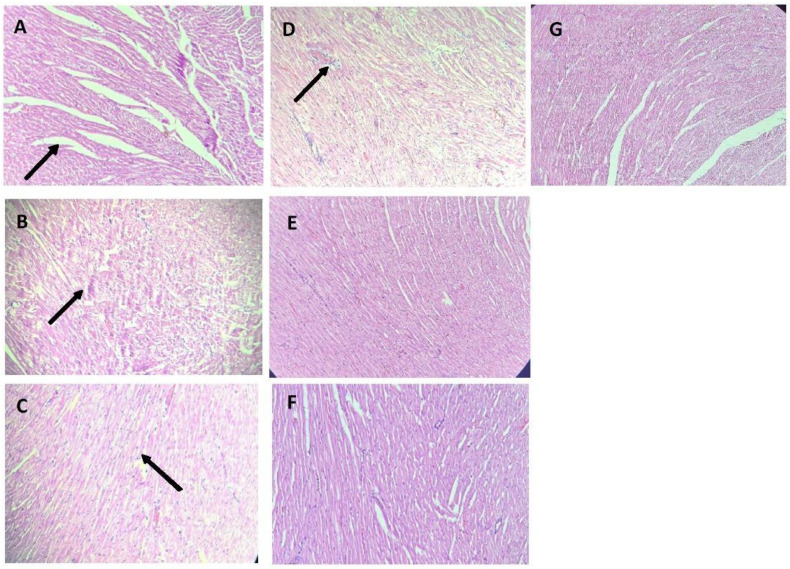
Histopathological changes in cardiac muscle (H-E stain). (**A**) Normotensive group, normal cardiac. The arrow indicates the transverse striation of the longitudinal section of the cardiac muscle.; (**B**) L-NAME group, chronic inflammation; (**C**) L-NAME + Captopril group, inflammatory cells decreased inflammation; (**D**) L-NAME + Esmeralda group, inflammatory cells are observed, moderate inflammation; (**E**) L-NAME + Perla group, reduction in inflammation; (**F**) L-NAME + Perla + Esmeralda group, without histopathological changes; (**G**) L-NAME Esmeralda + Captopril group, inflammation relief. The arrows in (**B**–**D**), indicate the infiltration of proinflammatory cells.

**Figure 4:**
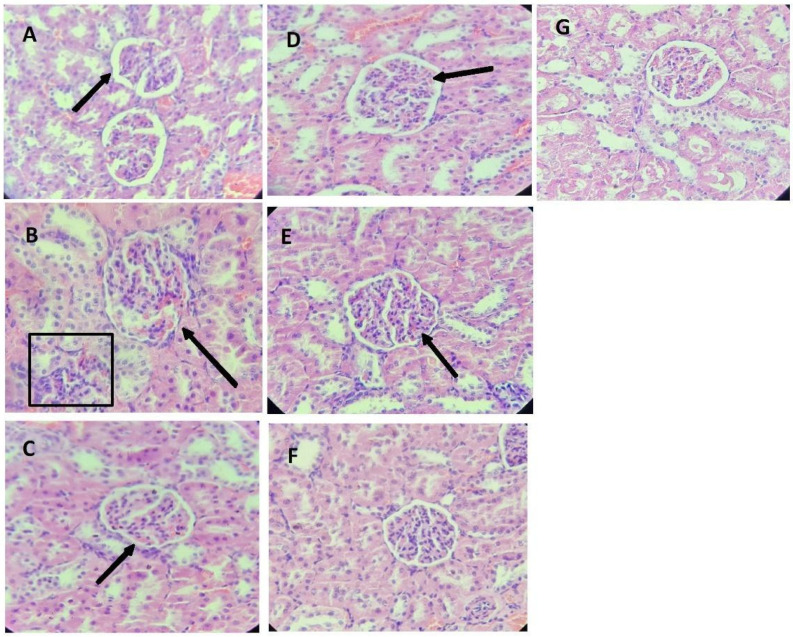
Histopathological changes in renal glomerulus. The arrows indicate the following changes in the renal glomerulus: (**A**) Normotensive group, without observable changes; (**B**) L-NAME group, degeneration and necrosis of renal glomerulus; (**C**) L-NAME + Captopril group, partial adhesion of glomerulus to Bowman’s capsules; (**D**) L-NAME + Esmeralda group, slight glomerular necrosis; (**E**) L-NAME + Perla group, Bowman space dilation; (**F**) L-NAME + Perla + Esmeralda group, decreased inflammation of renal glomerulus; (**G**) L-NAME Esmeralda + Captopril group, without observable histological changes.

Figure 3 and Figure 4 show the histopathologic changes in the heart and kidney of normotensive and L-NAME-induced hypertensive rats (G2). Figure 3A shows normal heart tissue with no signs of inflammation, while hypertensive rats exhibit myocardial fibrosis and mononuclear inflammatory cell infiltration, indicating chronic inflammation (Figure 3B). In contrast, treatment groups (G4, G5, G6, and G7) showed reduced muscle fibrosis and moderate myocardial degeneration (Figure 3C–G).

On the contrary, Figure 4A shows normal kidney histology, with intact renal glomeruli and clear Bowman’s capsules. L-NAME administration caused necrosis of the glomeruli, protein leakage, and dilation of Bowman’s, reducing fluid leakage (Figure 4B). Additionally, L-NAME treatment led to an increase in Kupffer cells in the liver, likely due to oxidative stress and inflammation (Table S1).

## 4. Discussion

The scientific evidence supporting the health benefits of germinated barley extracts (GBE) is emerging but still somewhat limited, particularly when it comes to in vivo and human studies. There is a solid foundation of in vitro and animal studies showing bioactivity related to antioxidant, anti-inflammatory, and antihypertensive effects, largely due to the increased presence of phenolic compounds and peptides during germination [20,31]. Germination is a promising process for developing novel nutritive and functional foods from barley with improved quality features. Additionally, germination can lead to a significant increase in the amino acids, protein, phenolic compounds, and antioxidative activity in Perla and Esmeralda barley extracts. More importantly, this can play an important role in reducing oxidative stress, a factor associated with hypertension [17,32]. Similarly, other studies have reported that sprouts of some legumes improve the quality and digestibility of starch and protein during germination [33]. In addition, the quality of barley sprouts has been studied in reducing the anti-inflammatory response in diseases such as fatty liver [34].

During germination, phenolic acids (e.g., gallic, syringic, and ferulic acid), flavonoids (e.g., catechin), and amino acids (e.g., valine and leucine) showed a linear correlation (r = 0.388 to 0.925) with the inhibition of ACE (Figure S1). In this study, Perla and Esmeralda’s SBEs showed inhibition between 56 and 63% and 59 and 83%, respectively. Contrastingly, Ra et al. (2020) reported ACE inhibition in barley between 32% and 66.5%, which they attributed to polyphenols such as 3-O-feruloyl quinic acid, saponarin, and orientin in the methanol extract of barley seedlings [35]. Based on the compound structure–activity relationships, the free hydroxyl groups of flavone-moieties and glucose connections at the ring of the flavone-moieties were important factors for the inhibition of ACE.

Interestingly, it has been demonstrated that certain phenolic compounds, such as catechins, may inhibit the activity of angiotensin-converting enzyme (ACE) by interacting with its active site. Specifically, catechins are capable of binding to the Zn(II) ion located in the catalytic domain of ACE, which is crucial for the enzyme’s function. This interaction is further stabilized by additional interactions with the amino acid residues present in the active site pockets, facilitated through hydrogen bonding and hydrophobic interactions. These combined interactions enhance the inhibitory potential of catechins, preventing the conversion of angiotensin I to angiotensin II thus contributing to their antihypertensive effect [29,36].

Results from this study showed a significant reduction in ACE I and II activities in both plasma and renal tissues following the administration of SBE, particularly of the Esmeralda variety. These findings are in agreement with previous reports that have demonstrated the ability of barley extracts, rich in bioactive compounds such as phenolic acids, flavonoids, and peptides, to inhibit ACE activity and thus reduce blood pressure. In the current study, Esmeralda showed a 63.3% inhibition of ACE I in plasma, while the combination of Esmeralda and the halved dose of captopril reduced ACE I activity by 81.6%, comparable to captopril alone (80.8%). This indicates a synergistic effect between SBE and conventional antihypertensive drugs such as captopril, supporting the hypothesis that bioactive compounds in barley can complement pharmacological treatments. Similar trends were observed for ACE II activity in renal tissue, where SBE treatments achieved a 76.5% to 80% reduction, restoring ACE II levels close to non-hypertensive controls. In this regard, Kumar et al. (2011) also showed that plant-derived peptides, including those from sprouted grains, could inhibit ACE and improve blood pressure regulation in hypertensive rats [30]. L-NAME inhibits all isoforms of NOS, reducing the synthesis of NO from L-arginine. NO is essential for endothelial function, promoting vasodilation, and maintaining normal blood vessel tone. Nitric oxide metabolites (NOx) play a crucial role in vascular function by promoting vasodilatation. In hypertension, oxidative stress reduces the bioavailability of NOx, leading to endothelial dysfunction and increasing vascular resistance. This dysregulation contributes to the development and maintenance of hypertension [37]. The absence of NO shifts the balance toward vasoconstriction, raising systemic vascular resistance and, consequently, blood pressure. In the evaluation of systolic and diastolic pressure, a significant decrease in systolic blood pressure (SBP) and diastolic blood pressure (DBP) was observed after the administration of germinated barley extracts. Germinated barley extracts, particularly from the Perla and Esmeralda varieties, showed a reduction in SBP of up to 12–17% and 10–17% in DBP, depending on the treatment time and the dose used, with a significant decrease in the SBE of each of the varieties separately (G4, G5), and with the combination G7. This decrease was comparable to that induced by captopril at standard doses. When comparing these results with other previous studies, it has been reported that barley extracts (not germinated) can also reduce blood pressure, although to a lesser extent. In this regard, a study by Ahmed-Farid et al. (2023) on the efficacy of barley ethanolic extract against a high salt diet (HSD)-induced cerebellum injury in hypertensive rats reported a decrease in blood pressure, but did not reach the levels of reduction observed with amlodipine used as a control [38].

The evaluation of renal function and nitric oxide (NOx) concentration following the administration of sprouted barley extracts (SBE) offers important insights into their potential nephroprotective effects, particularly in hypertensive conditions induced by L-NAME. The results presented indicate significant improvements in renal markers (creatinine, urea, and uric acid levels) and increased NOx levels, which are crucial for maintaining endothelial function. High plasma urea levels often indicate kidney dysfunction, which is a major contributor to volume-dependent hypertension due to impaired sodium and water excretion [39]. Uric acid has demonstrated a crucial role in the pathogenesis of hypertension and kidney disease progression. Possible pathophysiological mechanisms involve RAAS upregulation, kidney afferent arteriolopathy, endothelial dysfunction, oxidative stress, and systemic inflammation, which collectively exacerbate blood pressure [40]. Elevated serum creatinine reflects impaired renal function, which is a common cause and consequence of hypertension. Renal dysfunction disrupts the regulation of blood pressure by impairing sodium balance and promoting RAAS activation. Monitoring creatinine levels helps assess the extent of hypertensive damage to the kidneys [41]. In this study, creatinine levels, which had risen by 21% due to L-NAME, were reduced by 55% after treatment with Esmeralda SBE, showing a protective effect on renal function. The reduction in urea and uric acid levels in the treated groups also supports the beneficial role of SBE bioactives in improving renal health. Additionally, the rise in NOx levels by 67% in group G7 (SBE–captopril combination) highlights the ability of barley extracts to counteract the endothelial dysfunction caused by L-NAME-induced hypertension. The reversal of NOx depletion is consistent with previous findings on flavonoids and phenolic compounds in barley extracts, which have shown antioxidant and anti-inflammatory properties that protect kidney function and promote NO production. Comparatively, studies in vitro with barley sprout extracts (BSE) on inducible nitric oxide synthase (iNOS) showed potent anti-inflammatory activity in LPS-stimulated RAW 264.7 cells and particularly when subjected to fermentation with lactic acid bacteria. BSE suppressed LPS-induced production of NO, and this was accompanied by a decrease in the expression of the iNOS and COX-2 proteins [34,42,43]. Additionally, flavonoids and phenolic acids like quercetin are known to prevent NADPH oxidase expression, reduce ROS production, and positively impact NOx bioavailability, further influencing blood pressure regulation [44,45,46]. Similarly, other compounds in barley derived from tocopherols, such as polyprenols, can reduce oxidative stress and, due to their chemical composition, function as fat-soluble antioxidants [47,48].

Finally, the observed myocardial fibrosis, together with inflammatory cell infiltration, is a typical marker of chronic inflammation and cardiac remodeling occurring in induced hypertension models [49]. Glomerular necrosis and the dilation of Bowman spaces are also classic features of severe hypertension, where oxidative stress and inflammation play a critical role [50]. Likewise, the renal protection observed in SBE-treated groups (G4–G7), which included decreased necrosis and improved glomerular structure, was due to their higher content of antioxidants. Seven-day germination has been found to increase the total phenolic and flavonoid content by up to 285% and 316%, respectively [17]. However, the relationship between treatment duration and dose still needs to be further explored to maximize therapeutic benefits.

## 5. Conclusions

In conclusion, Perla and Esmeralda barley sprout extracts showed potent ACE inhibition (up to 83% in vitro). When combined with captopril, systolic blood pressure decreased by 22%, and ACE I and II activities were reduced by 81% and 76.5%, respectively. These extracts also improved renal function and elevated plasma NOx levels in hypertensive rats. Histopathological analysis indicates that SBE protects against heart, kidney, and liver tissue damage. The in vivo results obtained in this study suggest that SBE may be effective, particularly in combination, in reducing ACE activity, highlighting its potential as a nutraceutical intervention for hypertension. Despite growing evidence of health benefits, studies on sprouted barley extracts from the varieties studied are still limited, and further research is required to validate their nutraceutical use.

## Figures and Tables

**Table 1:** Effect of barley extracts on glucose content, renal function (mg/dL), and NOx production in plasma of rats induced with L-NAME.

Group	Glucose	Total Protein	Creatinine	Uric Acid	Urea	NOx µMol/L
G1. Standard diet	108.72 ± 10.85 ^b^	6.71 ± 0.91 ^a^	1.11 ± 0.15 ^ab^	1.13 ± 0.15 ^b^	18.73 ± 1.89 ^b^	83.88 ± 8.50 ^a^
G2. L-NAME (40 mg/kg)	106.69 ± 6.02 ^b^	6.20 ± 0.72 ^a^	1.34 ± 0.19 ^a^	1.70 ± 0.02 ^a^	31.25 ± 2.19 ^a^	52.66 ± 4.03 ^b^
G3. L-NAME + Captopril (5 mg/kg)	131.65 ± 4.73 ^a^	6.33 ± 0.66 ^a^	0.71 ± 0.07 ^cd^	0.97 ± 0.07 ^b^	19.82 ± 1.93 ^b^	86.11 ± 7.53 ^a^
G4. L-NAME + E7 (500 mg/kg)	104.81 ± 4.70 ^b^	7.21 ± 0.78 ^a^	0.60 ± 0.08 ^d^	1.09 ± 0.11 ^b^	16.35 ± 0.89 ^b^	80.00 ± 8.09 ^a^
G5. L-NAME + P7 (500 mg/kg)	98.11 ± 10.36 ^b^	6.97 ± 0.67 ^a^	0.94 ± 0.11 ^bc^	1.05 ± 0.12 ^b^	18.26 ± 1.89 ^b^	79.16 ± 6.18 ^a^
G6. L-NAME + E7 + P7 (250 + 250 mg/kg)	140.95 ± 11.01 ª	6.00 ± 0.67 ª	0.80 ± 0.09 ^bcd^	0.98 ± 0.1 ^b^	17.71 ± 1.91 ^b^	78.75 ± 7.88 ^a^
G7. L-NAME + E7+ Captopril (250 mg/kg + 2.5 mg/kg)	108.78 ± 10.85 ^b^	6.02 ± 0.98 ^a^	0.83 ± 0.10 ^bcd^	1.00 ± 0.08 ^b^	16.30 ± 1.64 ^b^	87.91 ± 6.85 ^a^

Values represent the mean ± SD (*n* = 6 rats); E7: SBE Esmeralda variety 7 days of germination; P7: SBE Perla variety 7 days of germination. Different lowercase letters indicate significant differences (*p* < 0.05) when compared with groups (ANOVA followed by Tukey test).

**Table 2:** Hemodynamic effects of administration of germinated barley extracts in rats.

	Systolic Blood Pressure (mm/Hg)			Diastolic Blood Pressure (mm/Hg)		
Groups/Week	1st	3rd	5th	1st	3rd	5th
G1. Standard diet	167.15 ± 6.41 ^bc^	166.58 ± 6.39 ^c^	161.36 ± 13.10 ^d^	134.46 ± 2.18 ^abcd^	127.73 ± 11.27 ^c^	115.82 ± 13.74 ^c^
G2. L-NAME (40 mg/kg)	193.32 ± 12.17 ^a^	214.72 ± 9.69 ^a^	218.62 ± 9.46 ^a^	154.52 ± 10.96 ^a^	177.33 ± 14.30 ^a^	182.92 ± 17.71 ^a^
G3. L-NAME + Captopril (5 mg/kg)	167.03±10.05 ^bc^	178.11 ± 9.56 ^bc^	178.28 ± 12.72 ^cd^	127.81 ± 10.38 ^bcd^	145.22 ± 9.96 ^bc^	148.35 ± 14.37 ^b^
G4. L-NAME + E7 (500 mg/kg)	165.86 ± 10.14 ^bc^	192.17 ± 6.44 ^b^	198.97 ± 9.97 ^b^	125.72 ± 12.72 ^cd^	151.18 ± 10.19 ^b^	159.94 ± 13.07 ^b^
G5. L-NAME + P7 (500 mg/kg)	161.41 ± 14.44 ^bc^	189.30 ± 10.29 ^b^	190.02 ± 15.97 ^bc^	120.98 ± 18.60 ^d^	152.52 ± 11.14 ^b^	157.85 ± 11.56 ^b^
G6. L-NAME + E7 + P7 (250 + 250 mg/kg)	187.08 ± 9.11 ^a^	189.93 ± 11.80 ^b^	190.92 ± 11.68 ^bc^	149.39 ± 15.29 ^ab^	158.12 ± 12.86 ^b^	156.12 ± 11.31 ^b^
G7. L-NAME + E7+ Captopril (250 mg/kg + 2.5 mg/kg)	176.56 ± 9.52 ^bc^	167.37 ± 12.81 ^c^	179.92 ± 11.56 ^bc^	142.32 ± 17.12 ^abcd^	132.27 ± 10.97 ^cd^	142.4 ± 12.37 ^bc^

All experiments represent the average of the measurements, followed by the standard deviation (*n* = 6–8); E7: SBE Esmeralda variety 7 days of germination; P7: SBE Perla variety 7 days of germination. Different lowercase letters indicate significant differences (*p* < 0.05) between groups in each time period.

## Data Availability

Dataset is available on request from the authors.

## Supplementary Materials

The following supporting information can be downloaded at: https://www.mdpi.com/article/10.3390/metabo14120678/s1, Figure S1: Histopathological changes in the liver (H-E stain); Table S1: Correlation between ACE inhibition (%) and bioactive compounds in SBE.
