# Supplementary material for: Antihypertensive Effect of Perla and Esmeralda Barley (Hordeum vulgare L.) Sprouts in an Induction Model with L-NAME In Vivo
Source: Metabolites. 2024 Dec 3;14(12):678. doi: 10.3390/metabo14120678 (PMC11676126; doi:10.3390/metabo14120678)
Supplement: Supplementary file 1 [file metabolites-14-00678-s001.zip › metabolites-3320650-supplementary.pdf]

Supplementary material.

Figure S1

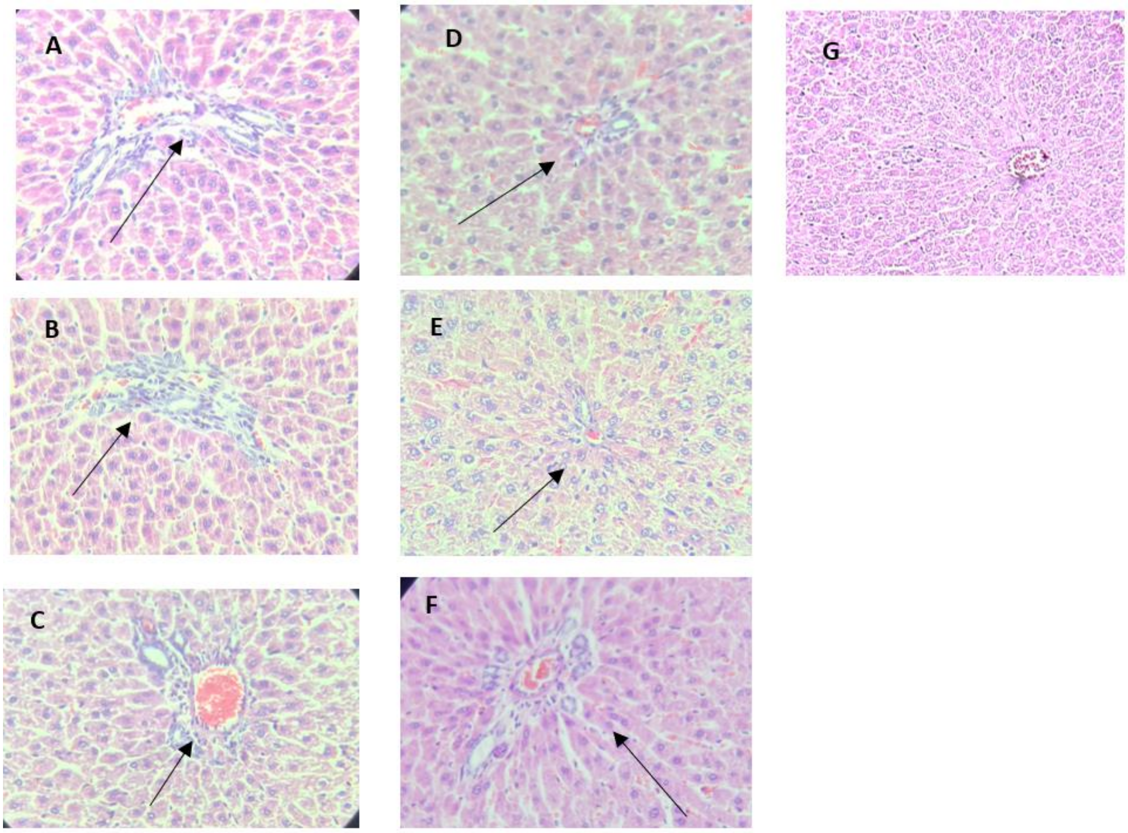

Figure S1. Histopathological changes in the liver (H-E stain). A) Normotensive group; B) L-NAME Group, increased Kupffer cell; C) L-NAME + Captopril Group, inflammatory cells decreased inflammation; D) L-NAME + Esmeralda Group, Kupffer cells are observed, high inflammation sign, moderate inflammation; E) L-NAME + Perla Group, reduction of inflammation; F) L-NAME + Perla + Esmeralda Group, mild inflammation; G) Group L-NAME Esmeralda + Captopril, no signs of inflammation

Supplementary material

Table S1

Correlation between ACE inhibition (%) and bioactive compounds in SBE.

Perla	**ACE inhibition (%) Correlation**
Catechin	0.693
Syringic acid	0.562
Isoleucine	0.751
Valine	0.388
Leucine	0.299
Esmeralda	
Catechin	0.925
Gallic acid	0.800
Ferulic acid	0.708
Coumaric acid	0.747
Syringic acid	0.630
Valine	0.906
Leucine	0.862
Isoleucine	0.831
